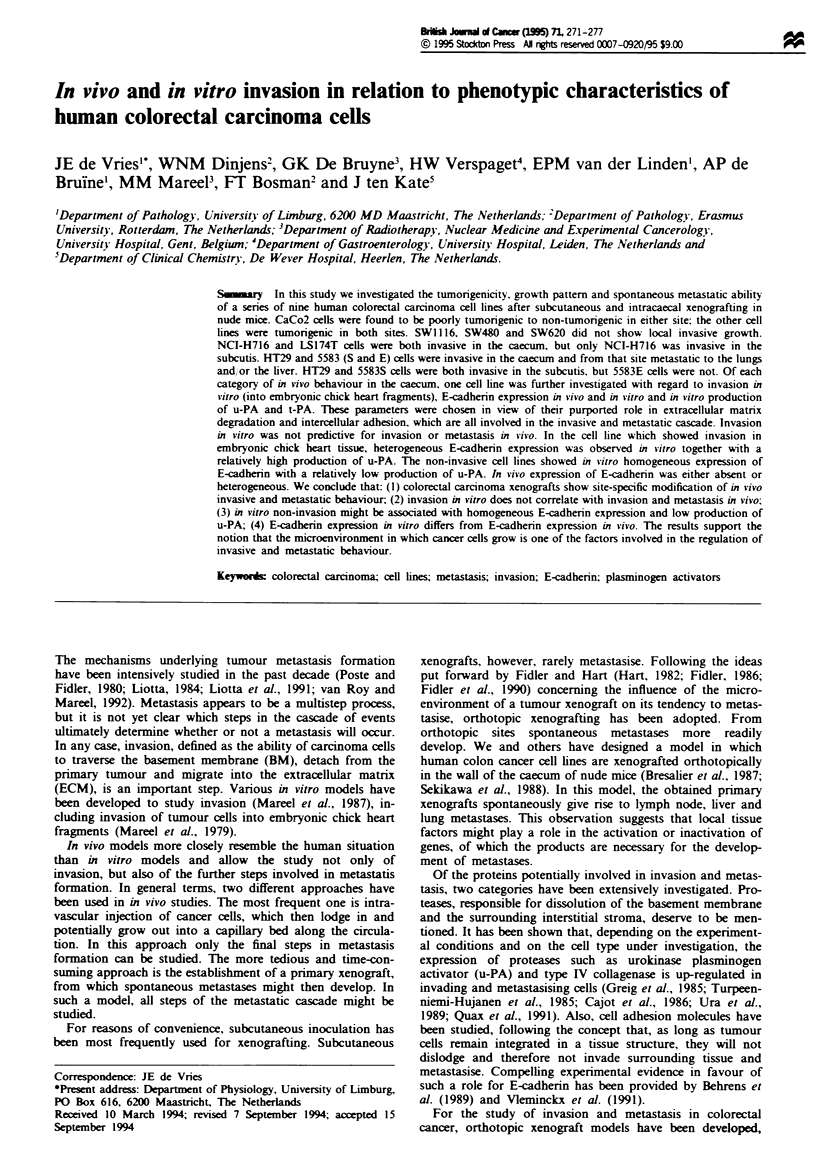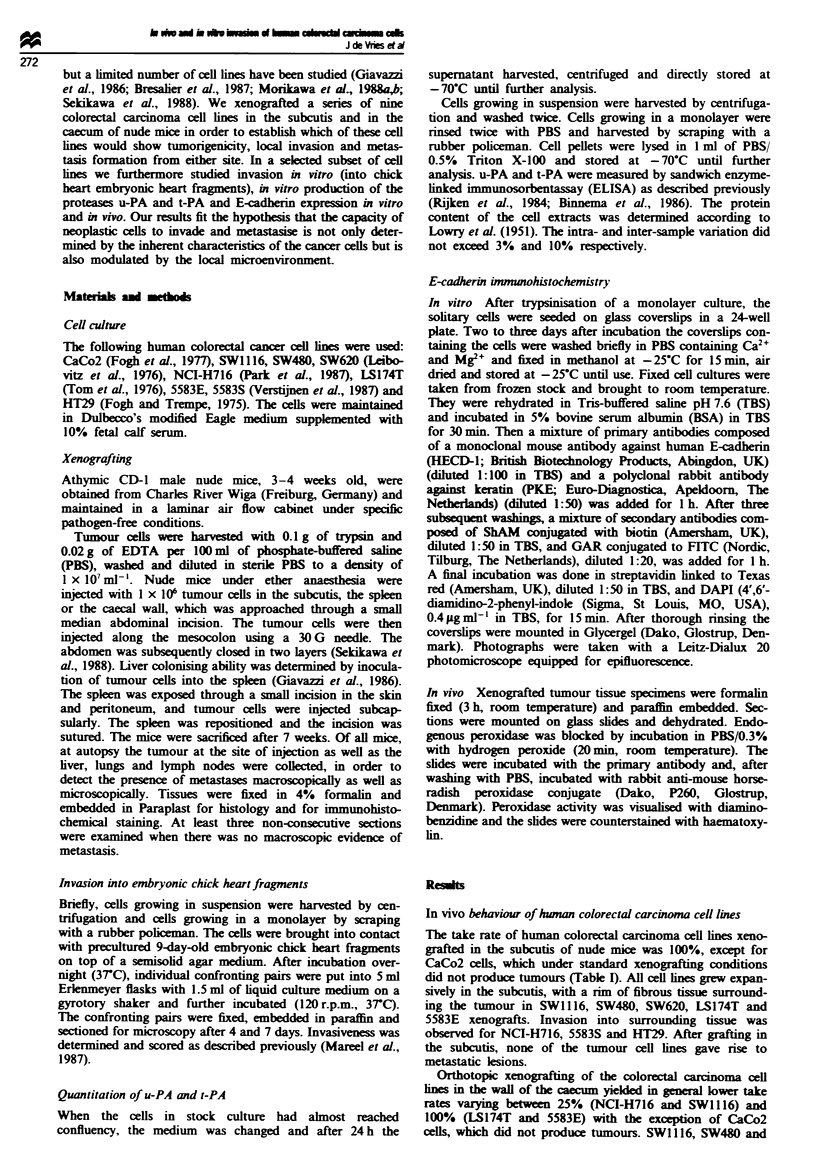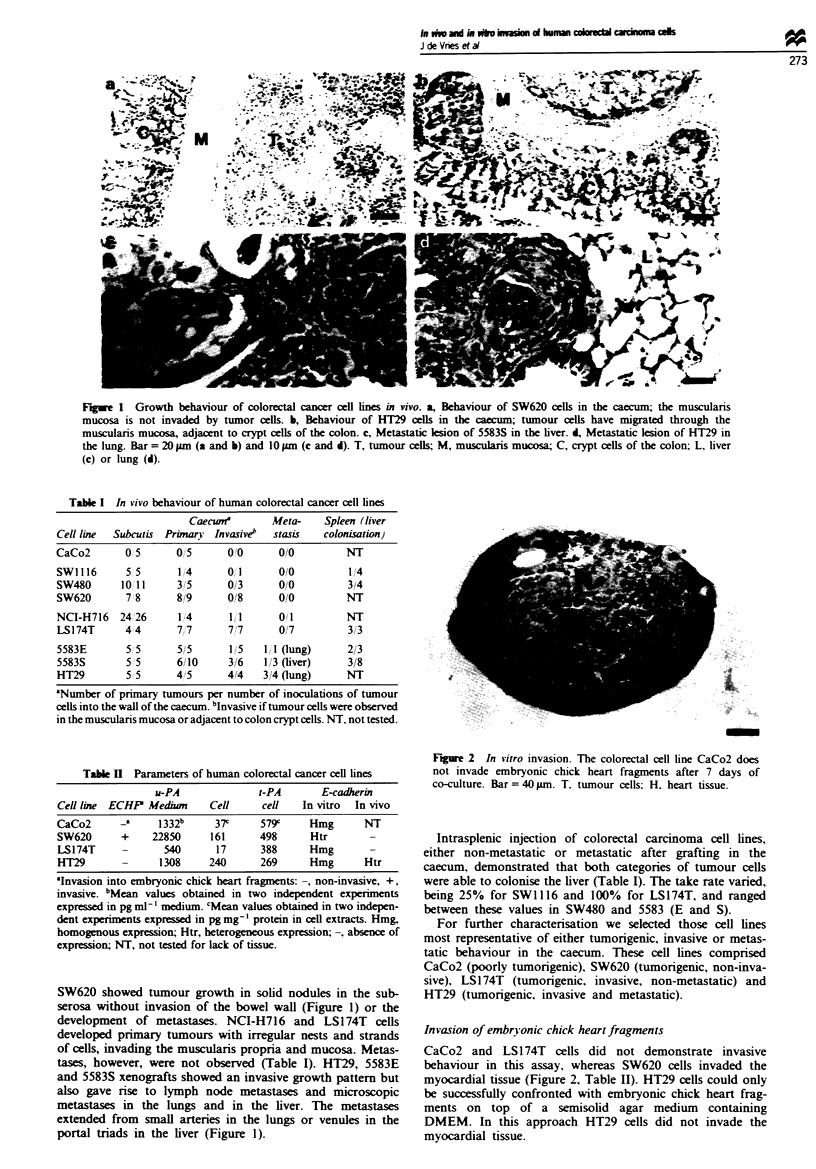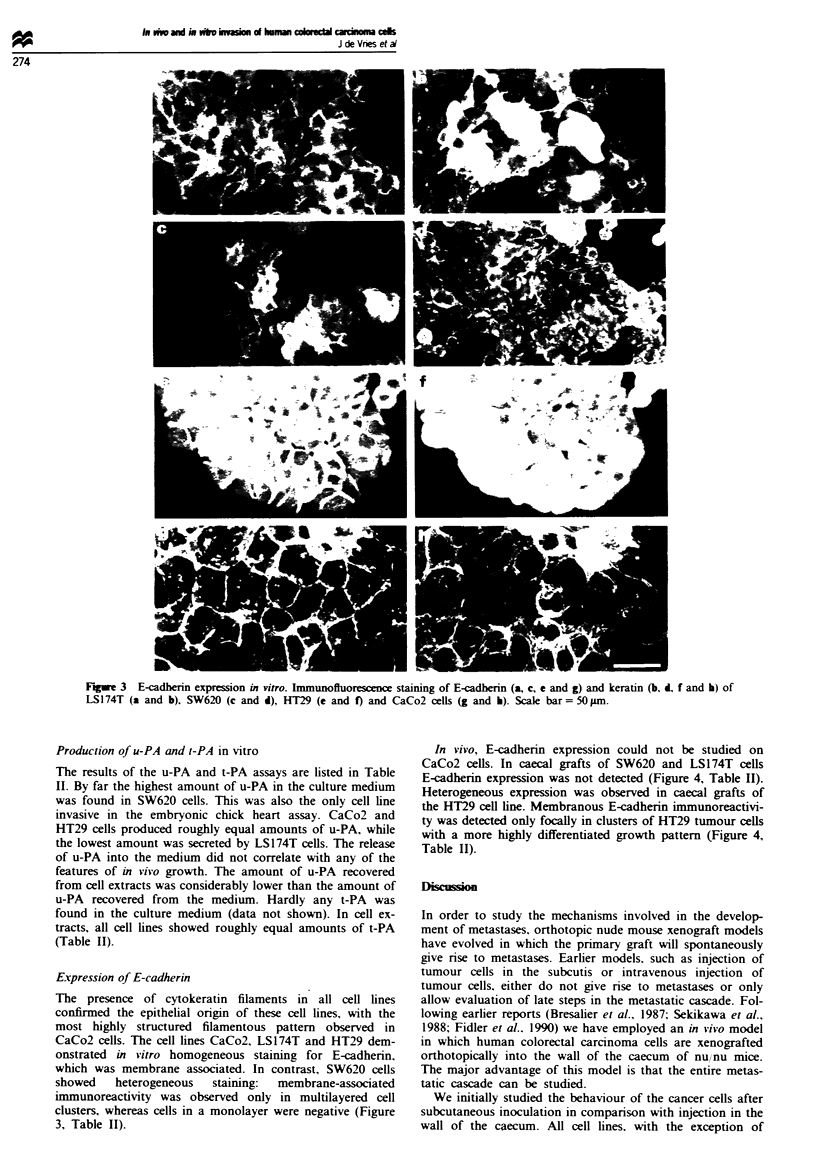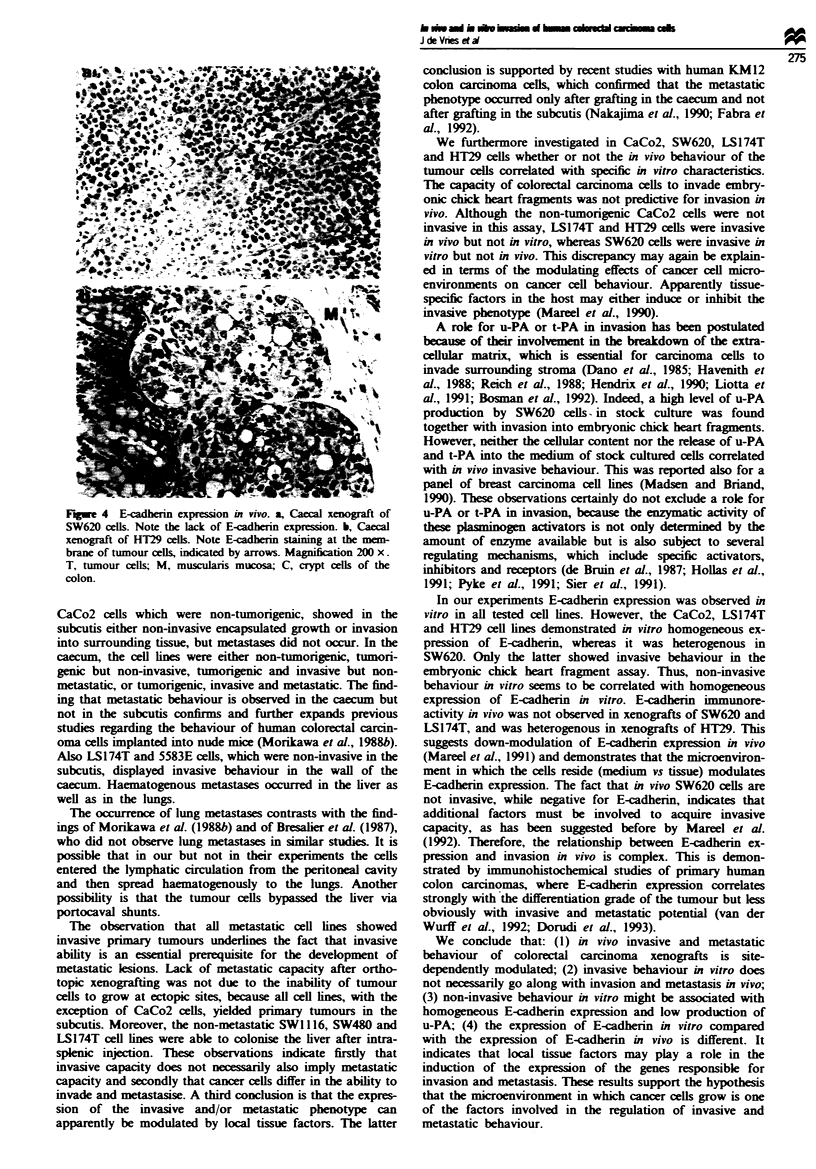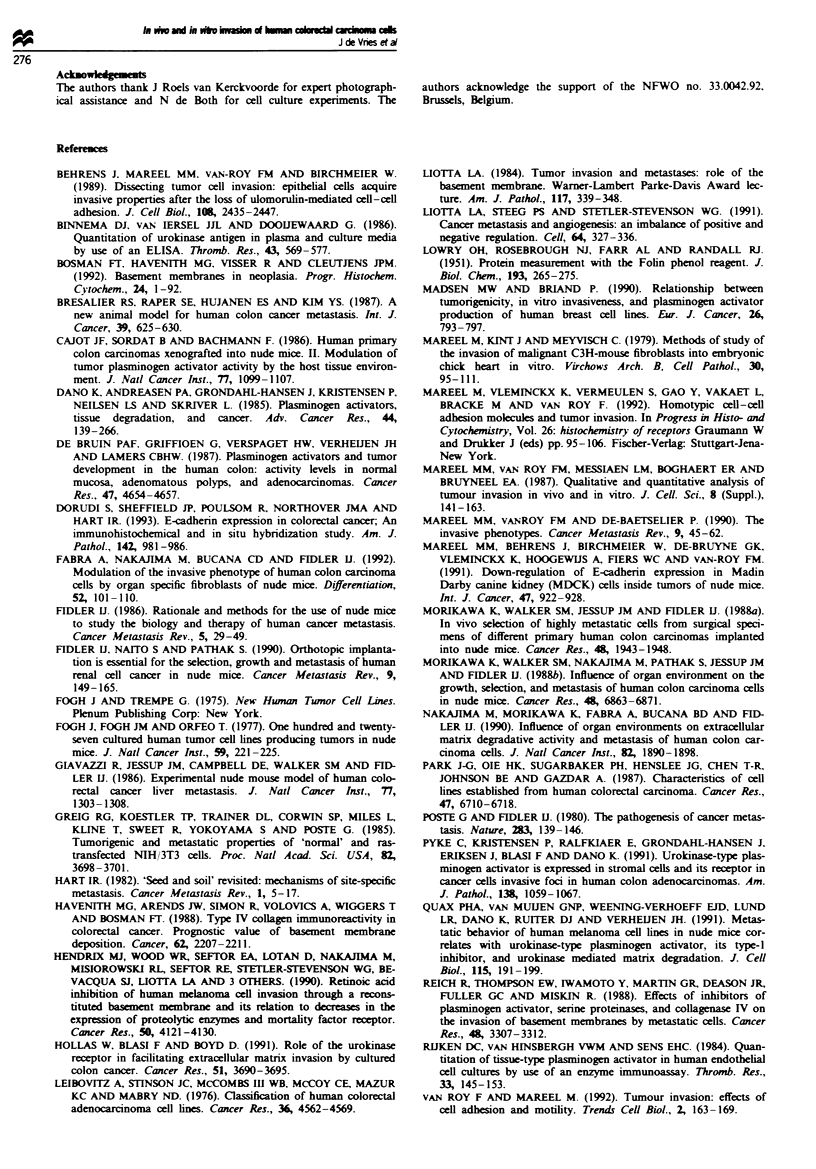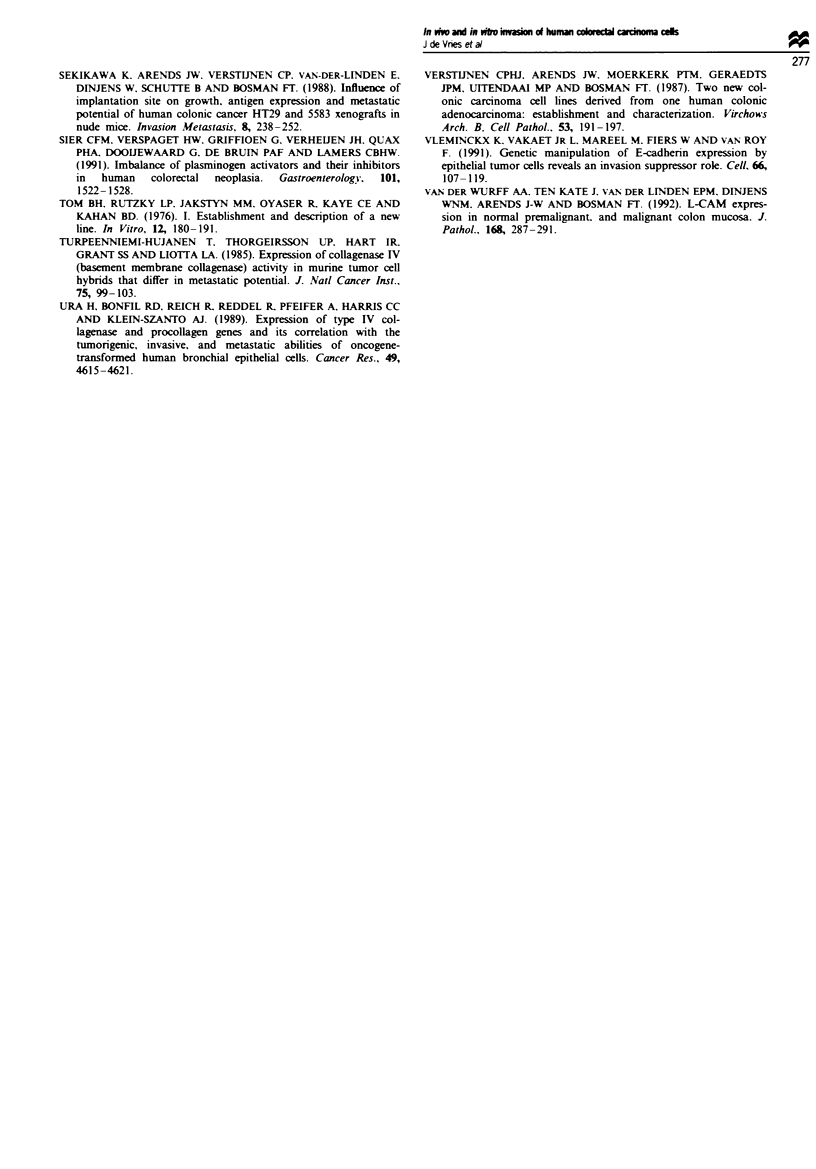# In vivo and in vitro invasion in relation to phenotypic characteristics of human colorectal carcinoma cells.

**DOI:** 10.1038/bjc.1995.55

**Published:** 1995-02

**Authors:** J. E. de Vries, W. N. Dinjens, G. K. De Bruyne, H. W. Verspaget, E. P. van der Linden, A. P. de Bruïne, M. M. Mareel, F. T. Bosman, J. ten Kate

**Affiliations:** Department of Pathology, University of Limburg, Maastricht, The Netherlands.

## Abstract

**Images:**


British Journal of Cancer (1995) 71, 271-277

© 1995 Stockton Press All rights reserved 0007-0920/95 $9.00

### *In vivo* and *in vitro* invasion in relation to phenotypic characteristics of human colorectal carcinoma cells

JE de Vries[1*], WNM Dinjens[2], GK De Bruyne[3], HW Verspaget[4], EPM van der Linden[1], AP de Bruïne[1], MM Mareel[3], FT Bosman[2] and J ten Kate[5]

[1]*Department of Pathology, University of Limburg, 6200 MD Maastricht, The Netherlands;* [2]*Department of Pathology, Erasmus University, Rotterdam, The Netherlands;* [3]*Department of Radiotherapy, Nuclear Medicine and Experimental Cancerology, University Hospital, Gent, Belgium;* [4]*Department of Gastroenterology, University Hospital, Leiden, The Netherlands and* [5]*Department of Clinical Chemistry, De Wever Hospital, Heerlen, The Netherlands.*

**Summary** In this study we investigated the tumorigenicity, growth pattern and spontaneous metastatic ability of a series of nine human colorectal carcinoma cell lines after subcutaneous and intracaecal xenografting in nude mice. CaCo2 cells were found to be poorly tumorigenic to non-tumorigenic in either site; the other cell lines were tumorigenic in both sites. SW1116, SW480 and SW620 did not show local invasive growth. NCI-H716 and LS174T cells were both invasive in the caecum, but only NCI-H716 was invasive in the subcutis. HT29 and 5583 (S and E) cells were invasive in the caecum and from that site metastatic to the lungs and or the liver. HT29 and 5583S cells were both invasive in the subcutis, but 5583E cells were not. Of each category of *in vivo* behaviour in the caecum, one cell line was further investigated with regard to invasion *in vitro* (into embryonic chick heart fragments), E-cadherin expression *in vivo* and *in vitro* and *in vitro* production of u-PA and t-PA. These parameters were chosen in view of their purported role in extracellular matrix degradation and intercellular adhesion, which are all involved in the invasive and metastatic cascade. Invasion *in vitro* was not predictive for invasion or metastasis *in vivo*. In the cell line which showed invasion in embryonic chick heart tissue, heterogeneous E-cadherin expression was observed *in vitro* together with a relatively high production of u-PA. The non-invasive cell lines showed *in vitro* homogeneous expression of E-cadherin with a relatively low production of u-PA. *In vivo* expression of E-cadherin was either absent or heterogeneous. We conclude that: (1) colorectal carcinoma xenografts show site-specific modification of *in vivo* invasive and metastatic behaviour; (2) invasion *in vitro* does not correlate with invasion and metastasis *in vivo*; (3) *in vitro* non-invasion might be associated with homogeneous E-cadherin expression and low production of u-PA; (4) E-cadherin expression *in vitro* differs from E-cadherin expression *in vivo*. The results support the notion that the microenvironment in which cancer cells grow is one of the factors involved in the regulation of invasive and metastatic behaviour.

**Keywords:** colorectal carcinoma; cell lines; metastasis; invasion; E-cadherin; plasminogen activators

The mechanisms underlying tumour metastasis formation have been intensively studied in the past decade (Poste and Fidler, 1980; Liotta, 1984; Liotta *et al.*, 1991; van Roy and Mareel, 1992). Metastasis appears to be a multistep process, but it is not yet clear which steps in the cascade of events ultimately determine whether or not a metastasis will occur. In any case, invasion, defined as the ability of carcinoma cells to traverse the basement membrane (BM), detach from the primary tumour and migrate into the extracellular matrix (ECM), is an important step. Various *in vitro* models have been developed to study invasion (Mareel *et al.*, 1987), including invasion of tumour cells into embryonic chick heart fragments (Mareel *et al.*, 1979).

*In vivo* models more closely resemble the human situation than *in vitro* models and allow the study not only of invasion, but also of the further steps involved in metastatis formation. In general terms, two different approaches have been used in *in vivo* studies. The most frequent one is intravascular injection of cancer cells, which then lodge in and potentially grow out into a capillary bed along the circulation. In this approach only the final steps in metastasis formation can be studied. The more tedious and time-consuming approach is the establishment of a primary xenograft, from which spontaneous metastases might then develop. In such a model, all steps of the metastatic cascade might be studied.

For reasons of convenience, subcutaneous inoculation has been most frequently used for xenografting. Subcutaneous xenografts, however, rarely metastasise. Following the ideas put forward by Fidler and Hart (Hart, 1982; Fidler, 1986; Fidler *et al.*, 1990) concerning the influence of the microenvironment of a tumour xenograft on its tendency to metastasise, orthotopic xenografting has been adopted. From orthotopic sites spontaneous metastases more readily develop. We and others have designed a model in which human colon cancer cell lines are xenografted orthotopically in the wall of the caecum of nude mice (Bresalier *et al.*, 1987; Sekikawa *et al.*, 1988). In this model, the obtained primary xenografts spontaneously give rise to lymph node, liver and lung metastases. This observation suggests that local tissue factors might play a role in the activation or inactivation of genes, of which the products are necessary for the development of metastases.

Of the proteins potentially involved in invasion and metastasis, two categories have been extensively investigated. Proteases, responsible for dissolution of the basement membrane and the surrounding interstitial stroma, deserve to be mentioned. It has been shown that, depending on the experimental conditions and on the cell type under investigation, the expression of proteases such as urokinase plasminogen activator (u-PA) and type IV collagenase is up-regulated in invading and metastasising cells (Greig *et al.*, 1985; Turpeenniemi-Hujanen *et al.*, 1985; Cajot *et al.*, 1986; Ura *et al.*, 1989; Quax *et al.*, 1991). Also, cell adhesion molecules have been studied, following the concept that, as long as tumour cells remain integrated in a tissue structure, they will not dislodge and therefore not invade surrounding tissue and metastasise. Compelling experimental evidence in favour of such a role for E-cadherin has been provided by Behrens *et al.* (1989) and Vleminckx *et al.* (1991).

For the study of invasion and metastasis in colorectal cancer, orthotopic xenograft models have been developed,

Correspondence: JE de Vries
*Present address: Department of Physiology, University of Limburg, PO Box 616, 6200 Maastricht, The Netherlands
Received 10 March 1994; revised 7 September 1994; accepted 15 September 1994

but a limited number of cell lines have been studied (Giavazzi *et al*., 1986; Bresalier *et al*., 1987; Morikawa *et al*., 1988*a*,*b*; Sekikawa *et al*., 1988). We xenografted a series of nine colorectal carcinoma cell lines in the subcutis and in the caecum of nude mice in order to establish which of these cell lines would show tumorigenicity, local invasion and metastasis formation from either site. In a selected subset of cell lines we furthermore studied invasion *in vitro* (into chick heart embryonic heart fragments), *in vitro* production of the proteases u-PA and t-PA and E-cadherin expression *in vitro* and *in vivo*. Our results fit the hypothesis that the capacity of neoplastic cells to invade and metastasise is not only determined by the inherent characteristics of the cancer cells but is also modulated by the local microenvironment.

### Materials and methods

### *Cell culture*

The following human colorectal cancer cell lines were used: CaCo2 (Fogh *et al*., 1977), SW1116, SW480, SW620 (Leibovitz *et al*., 1976), NCI-H716 (Park *et al*., 1987), LS174T (Tom *et al*., 1976), 5583E, 5583S (Verstijnen *et al*., 1987) and HT29 (Fogh and Trempe, 1975). The cells were maintained in Dulbecco's modified Eagle medium supplemented with 10% fetal calf serum.

### *Xenografting*

Athymic CD-1 male nude mice, 3–4 weeks old, were obtained from Charles River Wiga (Freiburg, Germany) and maintained in a laminar air flow cabinet under specific pathogen-free conditions.

Tumour cells were harvested with 0.1 g of trypsin and 0.02 g of EDTA per 100 ml of phosphate-buffered saline (PBS), washed and diluted in sterile PBS to a density of $1 \times 10^7$ $ml^{-1}$. Nude mice under ether anaesthesia were injected with $1 \times 10^6$ tumour cells in the subcutis, the spleen or the caecal wall, which was approached through a small median abdominal incision. The tumour cells were then injected along the mesocolon using a 30 G needle. The abdomen was subsequently closed in two layers (Sekikawa *et al*., 1988). Liver colonising ability was determined by inoculation of tumour cells into the spleen (Giavazzi *et al*., 1986). The spleen was exposed through a small incision in the skin and peritoneum, and tumour cells were injected subcapsularly. The spleen was repositioned and the incision was sutured. The mice were sacrificed after 7 weeks. Of all mice, at autopsy the tumour at the site of injection as well as the liver, lungs and lymph nodes were collected, in order to detect the presence of metastases macroscopically as well as microscopically. Tissues were fixed in 4% formalin and embedded in Paraplast for histology and for immunohistochemical staining. At least three non-consecutive sections were examined when there was no macroscopic evidence of metastasis.

### *Invasion into embryonic chick heart fragments*

Briefly, cells growing in suspension were harvested by centrifugation and cells growing in a monolayer by scraping with a rubber policeman. The cells were brought into contact with precultured 9-day-old embryonic chick heart fragments on top of a semisolid agar medium. After incubation overnight (37°C), individual confronting pairs were put into 5 ml Erlenmeyer flasks with 1.5 ml of liquid culture medium on a gyrotory shaker and further incubated (120 r.p.m., 37°C). The confronting pairs were fixed, embedded in paraffin and sectioned for microscopy after 4 and 7 days. Invasiveness was determined and scored as described previously (Mareel *et al*., 1987).

### *Quantitation of u-PA and t-PA*

When the cells in stock culture had almost reached confluency, the medium was changed and after 24 h the supernatant harvested, centrifuged and directly stored at −70°C until further analysis.

Cells growing in suspension were harvested by centrifugation and washed twice. Cells growing in a monolayer were rinsed twice with PBS and harvested by scraping with a rubber policeman. Cell pellets were lysed in 1 ml of PBS/0.5% Triton X-100 and stored at −70°C until further analysis. u-PA and t-PA were measured by sandwich enzyme-linked immunosorbentassay (ELISA) as described previously (Rijken *et al*., 1984; Binnema *et al*., 1986). The protein content of the cell extracts was determined according to Lowry *et al*. (1951). The intra- and inter-sample variation did not exceed 3% and 10% respectively.

### *E-cadherin immunohistochemistry*

*In vitro* After trypsinisation of a monolayer culture, the solitary cells were seeded on glass coverslips in a 24-well plate. Two to three days after incubation the coverslips containing the cells were washed briefly in PBS containing $Ca^{2+}$ and $Mg^{2+}$ and fixed in methanol at −25°C for 15 min, air dried and stored at −25°C until use. Fixed cell cultures were taken from frozen stock and brought to room temperature. They were rehydrated in Tris-buffered saline pH 7.6 (TBS) and incubated in 5% bovine serum albumin (BSA) in TBS for 30 min. Then a mixture of primary antibodies composed of a monoclonal mouse antibody against human E-cadherin (HECD-1; British Biotechnology Products, Abingdon, UK) (diluted 1:100 in TBS) and a polyclonal rabbit antibody against keratin (PKE; Euro-Diagnostica, Apeldoorn, The Netherlands) (diluted 1:50) was added for 1 h. After three subsequent washings, a mixture of secondary antibodies composed of ShAM conjugated with biotin (Amersham, UK), diluted 1:50 in TBS, and GAR conjugated to FITC (Nordic, Tilburg, The Netherlands), diluted 1:20, was added for 1 h. A final incubation was done in streptavidin linked to Texas red (Amersham, UK), diluted 1:50 in TBS, and DAPI (4′,6′-diamidino-2-phenyl-indole (Sigma, St Louis, MO, USA), 0.4 $\mu g\ ml^{-1}$ in TBS, for 15 min. After thorough rinsing the coverslips were mounted in Glycergel (Dako, Glostrup, Denmark). Photographs were taken with a Leitz-Dialux 20 photomicroscope equipped for epifluorescence.

*In vivo* Xenografted tumour tissue specimens were formalin fixed (3 h, room temperature) and paraffin embedded. Sections were mounted on glass slides and dehydrated. Endogenous peroxidase was blocked by incubation in PBS/0.3% with hydrogen peroxide (20 min, room temperature). The slides were incubated with the primary antibody and, after washing with PBS, incubated with rabbit anti-mouse horseradish peroxidase conjugate (Dako, P260, Glostrup, Denmark). Peroxidase activity was visualised with diaminobenzidine and the slides were counterstained with haematoxylin.

### Results

### In vivo *behaviour of human colorectal carcinoma cell lines*

The take rate of human colorectal carcinoma cell lines xenografted in the subcutis of nude mice was 100%, except for CaCo2 cells, which under standard xenografting conditions did not produce tumours (Table I). All cell lines grew expansively in the subcutis, with a rim of fibrous tissue surrounding the tumour in SW1116, SW480, SW620, LS174T and 5583E xenografts. Invasion into surrounding tissue was observed for NCI-H716, 5583S and HT29. After grafting in the subcutis, none of the tumour cell lines gave rise to metastatic lesions.

Orthotopic xenografting of the colorectal carcinoma cell lines in the wall of the caecum yielded in general lower take rates varying between 25% (NCI-H716 and SW1116) and 100% (LS174T and 5583E) with the exception of CaCo2 cells, which did not produce tumours. SW1116, SW480 and

**Figure 1** Growth behaviour of colorectal cancer cell lines *in vivo*. **a**, Behaviour of SW620 cells in the caecum; the muscularis mucosa is not invaded by tumor cells. **b**, Behaviour of HT29 cells in the caecum; tumour cells have migrated through the muscularis mucosa, adjacent to crypt cells of the colon. **c**, Metastatic lesion of 5583S in the liver. **d**, Metastatic lesion of HT29 in the lung. Bar = 20 μm (**a** and **b**) and 10 μm (**c** and **d**). T, tumour cells; M, muscularis mucosa; C, crypt cells of the colon; L, liver (**c**) or lung (**d**).

**Table I** *In vivo* behaviour of human colorectal cancer cell lines

| *Cell line* | *Subcutis* | *Caecum*[a] *Primary* | *Invasive*[b] | *Metastasis* | *Spleen (liver colonisation)* |
|---|---|---|---|---|---|
| CaCo2 | 0/5 | 0/5 | 0/0 | 0/0 | NT |
| SW1116 | 5/5 | 1/4 | 0/1 | 0/0 | 1/4 |
| SW480 | 10/11 | 3/5 | 0/3 | 0/0 | 3/4 |
| SW620 | 7/8 | 8/9 | 0/8 | 0/0 | NT |
| NCI-H716 | 24/26 | 1/4 | 1/1 | 0/1 | NT |
| LS174T | 4/4 | 7/7 | 7/7 | 0/7 | 3/3 |
| 5583E | 5/5 | 5/5 | 1/5 | 1/1 (lung) | 2/3 |
| 5583S | 5/5 | 6/10 | 3/6 | 1/3 (liver) | 3/8 |
| HT29 | 5/5 | 4/5 | 4/4 | 3/4 (lung) | NT |

[a]Number of primary tumours per number of inoculations of tumour cells into the wall of the caecum. [b]Invasive if tumour cells were observed in the muscularis mucosa or adjacent to colon crypt cells. NT, not tested.

**Figure 2** *In vitro* invasion. The colorectal cell line CaCo2 does not invade embryonic chick heart fragments after 7 days of co-culture. Bar = 40 μm. T, tumour cells; H, heart tissue.

**Table II** Parameters of human colorectal cancer cell lines

| *Cell line* | *ECHF*[a] | *u-PA Medium* | *u-PA Cell* | *t-PA cell* | *E-cadherin* In vitro | *E-cadherin* In vivo |
|---|---|---|---|---|---|---|
| CaCo2 | –[a] | 1332[b] | 37[c] | 579[c] | Hmg | NT |
| SW620 | + | 22850 | 161 | 498 | Htr | – |
| LS174T | – | 540 | 17 | 388 | Hmg | – |
| HT29 | – | 1308 | 240 | 269 | Hmg | Htr |

[a]Invasion into embryonic chick heart fragments: –, non-invasive, +, invasive. [b]Mean values obtained in two independent experiments expressed in pg $ml^{-1}$ medium. [c]Mean values obtained in two independent experiments expressed in pg $mg^{-1}$ protein in cell extracts. Hmg, homogenous expression; Htr, heterogeneous expression; –, absence of expression; NT, not tested for lack of tissue.

SW620 showed tumour growth in solid nodules in the subserosa without invasion of the bowel wall (Figure 1) or the development of metastases. NCI-H716 and LS174T cells developed primary tumours with irregular nests and strands of cells, invading the muscularis propria and mucosa. Metastases, however, were not observed (Table I). HT29, 5583E and 5583S xenografts showed an invasive growth pattern but also gave rise to lymph node metastases and microscopic metastases in the lungs and in the liver. The metastases extended from small arteries in the lungs or venules in the portal triads in the liver (Figure 1).

Intrasplenic injection of colorectal carcinoma cell lines, either non-metastatic or metastatic after grafting in the caecum, demonstrated that both categories of tumour cells were able to colonise the liver (Table I). The take rate varied, being 25% for SW1116 and 100% for LS174T, and ranged between these values in SW480 and 5583 (E and S).

For further characterisation we selected those cell lines most representative of either tumorigenic, invasive or metastatic behaviour in the caecum. These cell lines comprised CaCo2 (poorly tumorigenic), SW620 (tumorigenic, non-invasive), LS174T (tumorigenic, invasive, non-metastatic) and HT29 (tumorigenic, invasive and metastatic).

### *Invasion of embryonic chick heart fragments*

CaCo2 and LS174T cells did not demonstrate invasive behaviour in this assay, whereas SW620 cells invaded the myocardial tissue (Figure 2, Table II). HT29 cells could only be successfully confronted with embryonic chick heart fragments on top of a semisolid agar medium containing DMEM. In this approach HT29 cells did not invade the myocardial tissue.

**Figure 3** E-cadherin expression *in vitro*. Immunofluorescence staining of E-cadherin (**a**, **c**, **e** and **g**) and keratin (**b**, **d**, **f** and **h**) of LS174T (**a** and **b**), SW620 (**c** and **d**), HT29 (**e** and **f**) and CaCo2 cells (**g** and **h**). Scale bar = 50 μm.

*Production of u-PA and t-PA* in vitro

The results of the u-PA and t-PA assays are listed in Table II. By far the highest amount of u-PA in the culture medium was found in SW620 cells. This was also the only cell line invasive in the embryonic chick heart assay. CaCo2 and HT29 cells produced roughly equal amounts of u-PA, while the lowest amount was secreted by LS174T cells. The release of u-PA into the medium did not correlate with any of the features of *in vivo* growth. The amount of u-PA recovered from cell extracts was considerably lower than the amount of u-PA recovered from the medium. Hardly any t-PA was found in the culture medium (data not shown). In cell extracts, all cell lines showed roughly equal amounts of t-PA (Table II).

*Expression of E-cadherin*

The presence of cytokeratin filaments in all cell lines confirmed the epithelial origin of these cell lines, with the most highly structured filamentous pattern observed in CaCo2 cells. The cell lines CaCo2, LS174T and HT29 demonstrated *in vitro* homogeneous staining for E-cadherin, which was membrane associated. In contrast, SW620 cells showed heterogeneous staining: membrane-associated immunoreactivity was observed only in multilayered cell clusters, whereas cells in a monolayer were negative (Figure 3, Table II).

*In vivo*, E-cadherin expression could not be studied on CaCo2 cells. In caecal grafts of SW620 and LS174T cells E-cadherin expression was not detected (Figure 4, Table II). Heterogeneous expression was observed in caecal grafts of the HT29 cell line. Membranous E-cadherin immunoreactivity was detected only focally in clusters of HT29 tumour cells with a more highly differentiated growth pattern (Figure 4, Table II).

### Discussion

In order to study the mechanisms involved in the development of metastases, orthotopic nude mouse xenograft models have evolved in which the primary graft will spontaneously give rise to metastases. Earlier models, such as injection of tumour cells in the subcutis or intravenous injection of tumour cells, either do not give rise to metastases or only allow evaluation of late steps in the metastatic cascade. Following earlier reports (Bresalier *et al*., 1987; Sekikawa *et al*., 1988; Fidler *et al*., 1990) we have employed an *in vivo* model in which human colorectal carcinoma cells are xenografted orthotopically into the wall of the caecum of nu/nu mice. The major advantage of this model is that the entire metastatic cascade can be studied.

We initially studied the behaviour of the cancer cells after subcutaneous inoculation in comparison with injection in the wall of the caecum. All cell lines, with the exception of

**Figure 4** E-cadherin expression *in vivo*. **a**, Caecal xenograft of SW620 cells. Note the lack of E-cadherin expression. **b**, Caecal xenograft of HT29 cells. Note E-cadherin staining at the membrane of tumour cells, indicated by arrows. Magnification 200 ×. T, tumour cells; M, muscularis mucosa; C, crypt cells of the colon.

CaCo2 cells which were non-tumorigenic, showed in the subcutis either non-invasive encapsulated growth or invasion into surrounding tissue, but metastases did not occur. In the caecum, the cell lines were either non-tumorigenic, tumorigenic but non-invasive, tumorigenic and invasive but non-metastatic, or tumorigenic, invasive and metastatic. The finding that metastatic behaviour is observed in the caecum but not in the subcutis confirms and further expands previous studies regarding the behaviour of human colorectal carcinoma cells implanted into nude mice (Morikawa *et al*., 1988*b*). Also LS174T and 5583E cells, which were non-invasive in the subcutis, displayed invasive behaviour in the wall of the caecum. Haematogenous metastases occurred in the liver as well as in the lungs.

The occurrence of lung metastases contrasts with the findings of Morikawa *et al*. (1988*b*) and of Bresalier *et al*. (1987), who did not observe lung metastases in similar studies. It is possible that in our but not in their experiments the cells entered the lymphatic circulation from the peritoneal cavity and then spread haematogenously to the lungs. Another possibility is that the tumour cells bypassed the liver via portocaval shunts.

The observation that all metastatic cell lines showed invasive primary tumours underlines the fact that invasive ability is an essential prerequisite for the development of metastatic lesions. Lack of metastatic capacity after orthotopic xenografting was not due to the inability of tumour cells to grow at ectopic sites, because all cell lines, with the exception of CaCo2 cells, yielded primary tumours in the subcutis. Moreover, the non-metastatic SW1116, SW480 and LS174T cell lines were able to colonise the liver after intrasplenic injection. These observations indicate firstly that invasive capacity does not necessarily also imply metastatic capacity and secondly that cancer cells differ in the ability to invade and metastasise. A third conclusion is that the expression of the invasive and/or metastatic phenotype can apparently be modulated by local tissue factors. The latter conclusion is supported by recent studies with human KM12 colon carcinoma cells, which confirmed that the metastatic phenotype occurred only after grafting in the caecum and not after grafting in the subcutis (Nakajima *et al*., 1990; Fabra *et al*., 1992).

We furthermore investigated in CaCo2, SW620, LS174T and HT29 cells whether or not the *in vivo* behaviour of the tumour cells correlated with specific *in vitro* characteristics. The capacity of colorectal carcinoma cells to invade embryonic chick heart fragments was not predictive for invasion *in vivo*. Although the non-tumorigenic CaCo2 cells were not invasive in this assay, LS174T and HT29 cells were invasive *in vivo* but not *in vitro*, whereas SW620 cells were invasive *in vitro* but not *in vivo*. This discrepancy may again be explained in terms of the modulating effects of cancer cell microenvironments on cancer cell behaviour. Apparently tissue-specific factors in the host may either induce or inhibit the invasive phenotype (Mareel *et al*., 1990).

A role for u-PA or t-PA in invasion has been postulated because of their involvement in the breakdown of the extracellular matrix, which is essential for carcinoma cells to invade surrounding stroma (Dano *et al*., 1985; Havenith *et al*., 1988; Reich *et al*., 1988; Hendrix *et al*., 1990; Liotta *et al*., 1991; Bosman *et al*., 1992). Indeed, a high level of u-PA production by SW620 cells in stock culture was found together with invasion into embryonic chick heart fragments. However, neither the cellular content nor the release of u-PA and t-PA into the medium of stock cultured cells correlated with *in vivo* invasive behaviour. This was reported also for a panel of breast carcinoma cell lines (Madsen and Briand, 1990). These observations certainly do not exclude a role for u-PA or t-PA in invasion, because the enzymatic activity of these plasminogen activators is not only determined by the amount of enzyme available but is also subject to several regulating mechanisms, which include specific activators, inhibitors and receptors (de Bruin *et al*., 1987; Hollas *et al*., 1991; Pyke *et al*., 1991; Sier *et al*., 1991).

In our experiments E-cadherin expression was observed *in vitro* in all tested cell lines. However, the CaCo2, LS174T and HT29 cell lines demonstrated *in vitro* homogeneous expression of E-cadherin, whereas it was heterogenous in SW620. Only the latter showed invasive behaviour in the embryonic chick heart fragment assay. Thus, non-invasive behaviour *in vitro* seems to be correlated with homogeneous expression of E-cadherin *in vitro*. E-cadherin immunoreactivity *in vivo* was not observed in xenografts of SW620 and LS174T, and was heterogenous in xenografts of HT29. This suggests down-modulation of E-cadherin expression *in vivo* (Mareel *et al*., 1991) and demonstrates that the microenvironment in which the cells reside (medium *vs* tissue) modulates E-cadherin expression. The fact that *in vivo* SW620 cells are not invasive, while negative for E-cadherin, indicates that additional factors must be involved to acquire invasive capacity, as has been suggested before by Mareel *et al*. (1992). Therefore, the relationship between E-cadherin expression and invasion *in vivo* is complex. This is demonstrated by immunohistochemical studies of primary human colon carcinomas, where E-cadherin expression correlates strongly with the differentiation grade of the tumour but less obviously with invasive and metastatic potential (van der Wurff *et al*., 1992; Dorudi *et al*., 1993).

We conclude that: (1) *in vivo* invasive and metastatic behaviour of colorectal carcinoma xenografts is site-dependently modulated; (2) invasive behaviour *in vitro* does not necessarily go along with invasion and metastasis *in vivo*; (3) non-invasive behaviour *in vitro* might be associated with homogeneous E-cadherin expression and low production of u-PA; (4) the expression of E-cadherin *in vitro* compared with the expression of E-cadherin *in vivo* is different. It indicates that local tissue factors may play a role in the induction of the expression of the genes responsible for invasion and metastasis. These results support the hypothesis that the microenvironment in which cancer cells grow is one of the factors involved in the regulation of invasive and metastatic behaviour.

**Acknowledgements**

The authors thank J Roels van Kerckvoorde for expert photographical assistance and N de Both for cell culture experiments. The authors acknowledge the support of the NFWO no. 33.0042.92, Brussels, Belgium.

**References**

BEHRENS J, MAREEL MM, VAN-ROY FM AND BIRCHMEIER W. (1989). Dissecting tumor cell invasion: epithelial cells acquire invasive properties after the loss of ulomorulin-mediated cell-cell adhesion. *J. Cell Biol.*, **108**, 2435-2447.

BINNEMA DJ, VAN IERSEL JJL AND DOOIJEWAARD G. (1986). Quantitation of urokinase antigen in plasma and culture media by use of an ELISA. *Thromb. Res.*, **43**, 569-577.

BOSMAN FT, HAVENITH MG, VISSER R AND CLEUTJENS JPM. (1992). Basement membranes in neoplasia. *Progr. Histochem. Cytochem.*, **24**, 1-92.

BRESALIER RS, RAPER SE, HUJANEN ES AND KIM YS. (1987). A new animal model for human colon cancer metastasis. *Int. J. Cancer*, **39**, 625-630.

CAJOT JF, SORDAT B AND BACHMANN F. (1986). Human primary colon carcinomas xenografted into nude mice. II. Modulation of tumor plasminogen activator activity by the host tissue environment. *J. Natl Cancer Inst.*, **77**, 1099-1107.

DANO K, ANDREASEN PA, GRONDAHL-HANSEN J, KRISTENSEN P, NEILSEN LS AND SKRIVER L. (1985). Plasminogen activators, tissue degradation, and cancer. *Adv. Cancer Res.*, **44**, 139-266.

DE BRUIN PAF, GRIFFIOEN G, VERSPAGET HW, VERHEIJEN JH AND LAMERS CBHW. (1987). Plasminogen activators and tumor development in the human colon: activity levels in normal mucosa, adenomatous polyps, and adenocarcinomas. *Cancer Res.*, **47**, 4654-4657.

DORUDI S, SHEFFIELD JP, POULSOM R, NORTHOVER JMA AND HART IR. (1993). E-cadherin expression in colorectal cancer: An immunohistochemical and in situ hybridization study. *Am. J. Pathol.*, **142**, 981-986.

FABRA A, NAKAJIMA M, BUCANA CD AND FIDLER IJ. (1992). Modulation of the invasive phenotype of human colon carcinoma cells by organ specific fibroblasts of nude mice. *Differentiation*, **52**, 101-110.

FIDLER IJ. (1986). Rationale and methods for the use of nude mice to study the biology and therapy of human cancer metastasis. *Cancer Metastasis Rev.*, **5**, 29-49.

FIDLER IJ, NAITO S AND PATHAK S. (1990). Orthotopic implantation is essential for the selection, growth and metastasis of human renal cell cancer in nude mice. *Cancer Metastasis Rev.*, **9**, 149-165.

FOGH J AND TREMPE G. (1975). *New Human Tumor Cell Lines*. Plenum Publishing Corp: New York.

FOGH J, FOGH JM AND ORFEO T. (1977). One hundred and twenty-seven cultured human tumor cell lines producing tumors in nude mice. *J. Natl Cancer Inst.*, **59**, 221-225.

GIAVAZZI R, JESSUP JM, CAMPBELL DE, WALKER SM AND FIDLER IJ. (1986). Experimental nude mouse model of human colorectal cancer liver metastasis. *J. Natl Cancer Inst.*, **77**, 1303-1308.

GREIG RG, KOESTLER TP, TRAINER DL, CORWIN SP, MILES L, KLINE T, SWEET R, YOKOYAMA S AND POSTE G. (1985). Tumorigenic and metastatic properties of 'normal' and ras-transfected NIH 3T3 cells. *Proc. Natl Acad. Sci. USA*, **82**, 3698-3701.

HART IR. (1982). 'Seed and soil' revisited: mechanisms of site-specific metastasis. *Cancer Metastasis Rev.*, **1**, 5-17.

HAVENITH MG, ARENDS JW, SIMON R, VOLOVICS A, WIGGERS T AND BOSMAN FT. (1988). Type IV collagen immunoreactivity in colorectal cancer. Prognostic value of basement membrane deposition. *Cancer*, **62**, 2207-2211.

HENDRIX MJ, WOOD WR, SEFTOR EA, LOTAN D, NAKAJIMA M, MISIOROWSKI RL, SEFTOR RE, STETLER-STEVENSON WG, BEVACQUA SJ, LIOTTA LA AND 3 OTHERS. (1990). Retinoic acid inhibition of human melanoma cell invasion through a reconstituted basement membrane and its relation to decreases in the expression of proteolytic enzymes and mortality factor receptor. *Cancer Res.*, **50**, 4121-4130.

HOLLAS W, BLASI F AND BOYD D. (1991). Role of the urokinase receptor in facilitating extracellular matrix invasion by cultured colon cancer. *Cancer Res.*, **51**, 3690-3695.

LEIBOVITZ A, STINSON JC, MCCOMBS III WB, MCCOY CE, MAZUR KC AND MABRY ND. (1976). Classification of human colorectal adenocarcinoma cell lines. *Cancer Res.*, **36**, 4562-4569.

LIOTTA LA. (1984). Tumor invasion and metastases: role of the basement membrane. Warner-Lambert Parke-Davis Award lecture. *Am. J. Pathol.*, **117**, 339-348.

LIOTTA LA, STEEG PS AND STETLER-STEVENSON WG. (1991). Cancer metastasis and angiogenesis: an imbalance of positive and negative regulation. *Cell*, **64**, 327-336.

LOWRY OH, ROSEBROUGH NJ, FARR AL AND RANDALL RJ. (1951). Protein measurement with the Folin phenol reagent. *J. Biol. Chem.*, **193**, 265-275.

MADSEN MW AND BRIAND P. (1990). Relationship between tumorigenicity, in vitro invasiveness, and plasminogen activator production of human breast cell lines. *Eur. J. Cancer*, **26**, 793-797.

MAREEL M, KINT J AND MEYVISCH C. (1979). Methods of study of the invasion of malignant C3H-mouse fibroblasts into embryonic chick heart in vitro. *Virchows Arch. B, Cell Pathol.*, **30**, 95-111.

MAREEL M, VLEMINCKX K, VERMEULEN S, GAO Y, VAKAET L, BRACKE M AND VAN ROY F. (1992). Homotypic cell-cell adhesion molecules and tumor invasion. In *Progress in Histo- and Cytochemistry*, Vol. 26: *histochemistry of receptors* Graumann W and Drukker J (eds) pp. 95-106. Fischer-Verlag: Stuttgart-Jena-New York.

MAREEL MM, VAN ROY FM, MESSIAEN LM, BOGHAERT ER AND BRUYNEEL EA. (1987). Qualitative and quantitative analysis of tumour invasion in vivo and in vitro. *J. Cell. Sci.*, **8** (Suppl.), 141-163.

MAREEL MM, VANROY FM AND DE-BAETSELIER P. (1990). The invasive phenotypes. *Cancer Metastasis Rev.*, **9**, 45-62.

MAREEL MM, BEHRENS J, BIRCHMEIER W, DE-BRUYNE GK, VLEMINCKX K, HOOGEWIJS A, FIERS WC AND VAN-ROY FM. (1991). Down-regulation of E-cadherin expression in Madin Darby canine kidney (MDCK) cells inside tumors of nude mice. *Int. J. Cancer*, **47**, 922-928.

MORIKAWA K, WALKER SM, JESSUP JM AND FIDLER IJ. (1988*a*). In vivo selection of highly metastatic cells from surgical specimens of different primary human colon carcinomas implanted into nude mice. *Cancer Res.*, **48**, 1943-1948.

MORIKAWA K, WALKER SM, NAKAJIMA M, PATHAK S, JESSUP JM AND FIDLER IJ. (1988*b*). Influence of organ environment on the growth, selection, and metastasis of human colon carcinoma cells in nude mice. *Cancer Res.*, **48**, 6863-6871.

NAKAJIMA M, MORIKAWA K, FABRA A, BUCANA BD AND FIDLER IJ. (1990). Influence of organ environments on extracellular matrix degradative activity and metastasis of human colon carcinoma cells. *J. Natl Cancer Inst.*, **82**, 1890-1898.

PARK J-G, OIE HK, SUGARBAKER PH, HENSLEE JG, CHEN T-R, JOHNSON BE AND GAZDAR A. (1987). Characteristics of cell lines established from human colorectal carcinoma. *Cancer Res.*, **47**, 6710-6718.

POSTE G AND FIDLER IJ. (1980). The pathogenesis of cancer metastasis. *Nature*, **283**, 139-146.

PYKE C, KRISTENSEN P, RALFKIAER E, GRONDAHL-HANSEN J, ERIKSEN J, BLASI F AND DANO K. (1991). Urokinase-type plasminogen activator is expressed in stromal cells and its receptor in cancer cells invasive foci in human colon adenocarcinomas. *Am. J. Pathol.*, **138**, 1059-1067.

QUAX PHA, VAN MUIJEN GNP, WEENING-VERHOEFF EJD, LUND LR, DANO K, RUITER DJ AND VERHEIJEN JH. (1991). Metastatic behavior of human melanoma cell lines in nude mice correlates with urokinase-type plasminogen activator, its type-1 inhibitor, and urokinase mediated matrix degradation. *J. Cell Biol.*, **115**, 191-199.

REICH R, THOMPSON EW, IWAMOTO Y, MARTIN GR, DEASON JR, FULLER GC AND MISKIN R. (1988). Effects of inhibitors of plasminogen activator, serine proteinases, and collagenase IV on the invasion of basement membranes by metastatic cells. *Cancer Res.*, **48**, 3307-3312.

RIJKEN DC, VAN HINSBERGH VWM AND SENS EHC. (1984). Quantitation of tissue-type plasminogen activator in human endothelial cell cultures by use of an enzyme immunoassay. *Thromb. Res.*, **33**, 145-153.

VAN ROY F AND MAREEL M. (1992). Tumour invasion: effects of cell adhesion and motility. *Trends Cell Biol.*, **2**, 163-169.

SEKIKAWA K, ARENDS JW, VERSTIJNEN CP, VAN-DER-LINDEN E, DINJENS W, SCHUTTE B AND BOSMAN FT. (1988). Influence of implantation site on growth, antigen expression and metastatic potential of human colonic cancer HT29 and 5583 xenografts in nude mice. *Invasion Metastasis*, **8**, 238-252.

SIER CFM, VERSPAGET HW, GRIFFIOEN G, VERHEIJEN JH, QUAX PHA, DOOIJEWAARD G, DE BRUIN PAF AND LAMERS CBHW. (1991). Imbalance of plasminogen activators and their inhibitors in human colorectal neoplasia. *Gastroenterology*, **101**, 1522-1528.

TOM BH, RUTZKY LP, JAKSTYN MM, OYASER R, KAYE CE AND KAHAN BD. (1976). I. Establishment and description of a new line. *In Vitro*, **12**, 180-191.

TURPEENNIEMI-HUJANEN T, THORGEIRSSON UP, HART IR, GRANT SS AND LIOTTA LA. (1985). Expression of collagenase IV (basement membrane collagenase) activity in murine tumor cell hybrids that differ in metastatic potential. *J. Natl Cancer Inst.*, **75**, 99-103.

URA H, BONFIL RD, REICH R, REDDEL R, PFEIFER A, HARRIS CC AND KLEIN-SZANTO AJ. (1989). Expression of type IV collagenase and procollagen genes and its correlation with the tumorigenic, invasive, and metastatic abilities of oncogene-transformed human bronchial epithelial cells. *Cancer Res.*, **49**, 4615-4621.

VERSTIJNEN CPHJ, ARENDS JW, MOERKERK PTM, GERAEDTS JPM, UITENDAAI MP AND BOSMAN FT. (1987). Two new colonic carcinoma cell lines derived from one human colonic adenocarcinoma: establishment and characterization. *Virchows Arch. B, Cell Pathol.*, **53**, 191-197.

VLEMINCKX K, VAKAET JR L, MAREEL M, FIERS W AND VAN ROY F. (1991). Genetic manipulation of E-cadherin expression by epithelial tumor cells reveals an invasion suppressor role. *Cell*, **66**, 107-119.

VAN DER WURFF AA, TEN KATE J, VAN DER LINDEN EPM, DINJENS WNM, ARENDS J-W AND BOSMAN FT. (1992). L-CAM expression in normal premalignant, and malignant colon mucosa. *J. Pathol.*, **168**, 287-291.